# Challenges in microbiological diagnosis of invasive *Aspergillus* infections

**DOI:** 10.12688/f1000research.10216.1

**Published:** 2017-02-17

**Authors:** Alexandre Alanio, Stéphane Bretagne

**Affiliations:** 1 Parasitology-Mycology Laboratory, Lariboisière Saint-Louis Fernand Widal hospitals, Assistance Publique-Hôpitaux de Paris (AP-HP), Paris, France; 2 Paris-Diderot, Sorbonne Paris Cité University, Paris, France; 3 Institut Pasteur, CNRS, Molecular Mycology Unit, Reference National Center of Invasive Mycoses and Antifungals, Paris, France

**Keywords:** Aspergillosis, diagnosis, at-risk patients, fungal identification

## Abstract

Invasive aspergillosis (IA) has been increasingly reported in populations other than the historical hematology patients and there are new questions about the performance of microbiological tools. Microscopy and culture have been completed by biomarkers, either antigens or DNA, and in blood or respiratory specimens or both. First studied in hematology, the antigen galactomannan performance in serum is low in other patient populations where the pathophysiology of the infection can be different and the prevalence of IA is much lower. DNA detection with polymerase chain reaction (PCR) in blood or serum (or both) has reached a certain level of acceptance thanks to consensus methods based on real-time quantitative PCR (qPCR). When used on respiratory specimens, galactomannan and qPCR depend on standardization of the sampling and the diverse mycological procedures. Thus, culture remains the main diagnostic criterion in critically ill patients. The current trend toward more effective anti-mold prophylaxis in hematology hampers the yield of a screening strategy, as is usually performed in hematology. Therefore, circulating biomarkers as confirmatory tests should be considered and their performance should be reappraised in each new setting. The use of azole prophylaxis also raises the issue of selecting azole-resistance *Aspergillus fumigatus* isolates. Ideally, the biomarkers will be more efficient when individual genetic risks of IA are defined. Culture, though not standardized, remains a key element for the diagnosis of IA and has the advantage to easily detect molds other than *A. fumigatus*. It is still unclear whether next-generation sequencing will replace culture in the future.

## Introduction

Invasive aspergillosis (IA) is the prototype of opportunistic diseases: all of the diagnostic and therapeutics difficulties are due to the fact that only the presence of the germ cannot identify the infection. To help epidemiological studies and the evaluation of therapeutic trials, the European Organization for Research and Treatment of Cancer/Invasive Fungal Infections Cooperative Group and the National Institute of Allergy and Infectious Diseases Mycoses Study Group (EORTC/MSG) proposed criteria for defining IA, incorporating clinical, imaging, and microbiological items ^1^. These definitions are suitable for hematological diseases—mainly, acute leukemia and hematopoietic stem cell transplant (HSCT) recipients—and, to a lesser extent, solid organ transplantations (SOTs). However, either because clinicians of other specialties are increasingly interested in IA or because of the improvement of diagnostic means, IA is now reported in other immunocompromised patient populations, such as intensive care unit (ICU) patients, patients given anti-tumor necrosis factor (anti-TNF) therapy ^2^, patients with AIDS ^3^, as well as in hematology, in patients with chronic lymphoproliferative diseases, including multiple myeloma ^4^ instead of acute leukemia ^5^. Definitions based on the EORTC/MSG criteria were not well adapted to these new populations associated with a low performance of serum galactomannan (GM) testing ^6^. The current challenges therefore are to adapt or develop new diagnostic microbiological criteria and to reassess the performance of biomarkers in these new populations ^7^.

In parallel, new therapeutic strategies (mainly, prophylaxis in hematology) are now widely used. If the expected result is a decrease of IA prevalence, a bystander effect, though also expected, is a negative impact on the performance of biomarkers, which must be reappraised ^8, 9^. Indeed, the prevalence of the disease directly impacts the performance of any test and GM in particular ^6^. In parallel, this extensive use of prophylaxis has also shed light on the occurrence of azole-resistant *Aspergillus fumigatus* isolates.

## New populations at risk of invasive aspergillosis

In hematology, IA is increasingly reported late after HSCT ^10^ and in chronic lymphoproliferative disorders, mainly after several therapeutic interventions ^5^. In these patients, the main risk factor of IA is not a profound and prolonged neutropenia as historically reported in acute leukemia, but instead high-dose steroid therapy ^5, 10^. When IA develops during steroid therapy, the pathology of the lesions shows little fungus and chronic inflammation and a lower chance that the antigen will reach the blood stream ^11^. This probably explains why GM is released in a lower quantity in serum when patients are not neutropenic ^12, 13^. The value of GM screening in chronic lymphoproliferative disorders therefore needs to be reassessed.

Another new group of patients who have been reported to be at risk of IA are the critically ill patients in an ICU without malignancies or other known risk factors for IA. High-dose corticosteroids and comorbidities such as chronic obstructive pulmonary disease, liver or renal failure, and diabetes are commonly noted as well as all causes of severe sepsis ^14– 17^. The issue underlined in these patients is the value of isolation of *Aspergillus* spp. from respiratory specimens given the absence of classic imaging signs such as a well-circumscribed nodule with halo or air crescent signs. A specific algorithm has recently been proposed to discriminate colonization from IA in critically ill patients with the introduction of “putative aspergillosis” as a new category ^16^. Thus, putative aspergillosis represented 38% of patients with a positive *Aspergillus* culture, which could represent a high number of ICU patients. The definition of putative aspergillosis relies on four criteria: an *Aspergillus*-positive lower respiratory tract specimen (entry condition); abnormal medical imaging; and either other host risk factors or a semi-quantitative *Aspergillus*-positive culture of bronchoalveolar lavage (BAL) fluid with a positive microscopy (hyphae with morphology indicative of *Aspergillus* sp.) and without bacterial growth ^16^. This would imply that the microbiological procedures to identify, quantify, and culture molds are similar in all microbiology laboratories, which is not the case even for hematology patients at high risk of IA ^18^. Additionally, this definition does not make any distinction between the *Aspergillus* species, although they do not all have the same virulence and anti-fungal susceptibility profile ^5^. Therefore, if the mycological criteria for defining IA in ICU patients are microscopy and isolation of *Aspergillus* spp., some consensus microbiological methods should be accepted.

In other patient populations such as patients who receive anti-TNF therapy ^2^ or patients with AIDS ^3^, the incidence seems to remain low, although surveillance is always warranted. Similarly, a better index of suspicion is needed for the localizations that are not pulmonary, such as intestinal localizations, which are always difficult to diagnose ^19^.

## Genetic susceptibility and environmental factors

Currently, the microbiological observations for the diagnosis of IA are analyzed among patients with a similar underlying disease with the understanding that all the patients with the same factors are similarly at risk. However, one has always observed differences between patients; some develop IA and others do not in the same environment and under the same treatment. This observation has been revisited with the current availability of large-scale genetic screening means. Some donor haplotypes in Toll-like receptor 4 were shown to be associated with an increased risk of IA in hematopoietic cell transplants from unrelated donors ^20^. More recently, single-nucleotide polymorphisms in the nuclear factor kappa B (NFκB)-related genes were not found to be associated with an increased risk of developing IA ^21^. Even if the current results are not very conclusive, this is an obvious field that needs to be investigated for a better assignment of anti-fungal drugs to patients at risk of IA. When this genetic susceptibility is better known, the meaning of the microbiological observation can be better interpreted.

Additionally, the environmental factors of the patients should be considered. Although IA occurs more often in outpatients, the different home or work environments are rarely investigated. There is now evidence that all patients do not share the same risk of inhaling pathogenic spores when returning home ^22^, yet this risk is rarely taken into consideration ^23, 24^.

## Diagnostic means

Besides direct microscopy and culture, which remain important for identification and anti-fungal susceptibility testing of the fungus, several biomarkers have been evaluated: GM, 1,3-B-D-glucan (BDG), and *Aspergillus* DNA. These biomarkers have been tested in blood or serum (or both) and in respiratory specimens. The main issue for the present circulating biomarkers is their weak specificity. This weak specificity is due to the analytical performance of the tests but also to the presence of the biomarkers tested in the environment, including food, inhaled air, pharmaceutical products ^25, 26^, or blood tubes ^27^.

Antibody detection has been poorly studied for IA because of the underlying immunodepression and is not considered a diagnostic criterion ^1^. Moreover, the assays are not standardized. Therefore, they will not be commented on here despite their major interest in chronic and allergic forms of pulmonary aspergillosis ^28^.

## Serum antigens: galactomannan and beta-D-glucan

GM remains a cornerstone for the diagnosis of IA, which is far ahead of BDG, whose specificity is poor because of the pan-fungal nature of this marker ^29, 30^. GM is a microbiological criterion for defining IA in hematology ^31^ and SOT ^32^ patients, but there are difficulties related to studying its performance as an evaluation tool and as a diagnostic criterion ^33^. GM is also used as a surrogate marker to follow the efficacy of treatment ^34^. The main limitation of serum GM is the high rate of false-positive results, which fall under two categories: irreproducible results and detection not related to IA. The irreproducible positive results correspond to results that are not confirmed when retested and should be considered negative ^35^. Confirmed positive results are technically true positive, which corresponds to either a true IA or the presence of GM from environmental sources ^25, 36^. There is currently no means to safely discard a GM-positive result that is not related to ongoing IA based on technical artifacts. Only the analysis of intravenous drugs or parenteral nutrition prescribed and possibly the test of batches of drugs thought to contain manufactured GM can support a false positive due to infused products, mainly antibiotics processed through mold cultures ^25^. Otherwise, it is the analysis of the evolution of the disease and medical file records that allow discrimination between true-positive or false-positive results.

Currently, the medical performance of GM is decreasing due to the widespread use of effective anti-mold prophylaxis ^8, 9^. In these conditions, the screening strategy usually proposed in hematology ^37^ could become inefficient and would no longer be recommended ^8, 9^. If it is used as confirmatory test, thresholds to define positivity could have to be reassessed for GM and also for other biomarkers. For instance, the GM positivity is 0.5 in two separate serum samples as used as a screening test, because the goals are to minimize the risk of IA and to limit the risk of over-treating, whereas the positivity threshold is at least 1.0 in BAL fluid sample to assess the diagnosis ( http://www.fda.gov/downloads/Drugs/GuidanceComplianceRegulatoryInformation/Guidances/UCM420248.pdf).

Recently, a lateral flow device (LFD) was compared with PCR and GM ^38^. This test is based on the detection of a new fungal antigen, an extracellular protein, using a monoclonal antibody. The best performance was achieved in combination with PCR, providing both 100% sensitivity and 100% specificity. Its main advantage should be to provide clinicians with a rapid operational result, even if PCR can be performed later as a confirmatory test.

## Polymerase chain reaction in blood/serum

The first publications on the detection of circulating *A. fumigatus* DNA appeared in the 1990s either in whole blood ^39^ or in serum ^40^. However, as soon as this occurred, PCR for IA was shown to be highly challenging because of the very low amount of DNA in samples. This low amount exacerbated all of the pitfalls and limits of the PCR, mainly false positives due to previously amplified products and environmental contamination and false-negative results due to residual PCR inhibitors ^41^. In 2006, the European Aspergillus PCR Initiative (EAPCRI) was launched to seek proposals for a technical consensus. This consensus was possible thanks to the generalization of real-time quantitative PCR (qPCR), which dramatically reduces the risk of contamination from environmental amplicons and allows quantitative management of the amplification reaction to detect inhibition ^41^. Because whole blood is technically more demanding for the extraction steps, serum appears to be a better specimen ^42^. More recently, plasma has been shown to have a better sensitivity than serum and should be the preferred specimen ^43^. The performance of PCR in blood seems at least as good as GM ^44^, and the absence of both biomarkers (that is, GM and circulating DNA) could be sufficient to postpone anti-fungal therapy ^45^. As for GM, it remains unclear whether PCR should be used as a screening test ^46^ or as a confirmatory test in light of the widespread use of anti-mold prophylaxis ^9^.

## Combined use of blood biomarkers

A recent meta-analysis focused on pediatric cancer and HSCT and analyzing the biomarkers (GM, BDG, and DNA) separately concluded a poor performance of the tests, and the authors suggested combining several biomarkers for further studies ^47^. As soon as PCR was developed for IA diagnosis, the interest of associating GM and DNA detection was evaluated ^40^. Another recent meta-analysis showed that the association of both tests is highly suggestive of an active infection with a positive predictive value of 88% ^45^. A therapeutic strategy based on a combined surveillance of serum GM and *Aspergillus* DNA was shown to decrease the incidence of IA in high-risk hematological patients ^46^. The parallel use of GM and PCR was effective in reducing empirical anti-fungal treatment in hematology patients at high risk of IA ^31^. The combined use of LFD, instead of GM, and qPCR seems could be a better strategy ^38^.

## Biomarkers in bronchoalveolar lavage fluids

Biomarkers in respiratory specimens are more prone to subjective interpretation than the same biomarkers in blood. Indeed, a positive mold culture from a respiratory specimen can be ascribed to infection, colonization, or simple bystander observation. To switch from culture to biomarkers does not radically change the issue. Additionally, biomarker assays are more amenable to standardization compared with the same assays in respiratory specimens.

GM has also been investigated in BAL fluids and some authors advocate the value of GM testing in the ICU ^48^. GM has also been evaluated in association with qPCR with the delineation of quantitative thresholds both for qPCR and for GM ^49^. However, to be widely accepted, these thresholds should be of similar values in different settings. Unfortunately, the BAL procedures are highly variable between centers (for example, three lavages of 50 mL versus two lavages of 20 mL); more importantly, they are highly variable between patients according to their underlying pulmonary lesions ^18^. Therefore, there are substantial difficulties in obtaining consensus for quantitative thresholds.

The LFD described above has also been evaluated in BAL fluids. When the LFD assay was used alone in BAL of patients with hematological malignancies, the sensitivity of the LFD was moderate, around 60% to 70%, which according to the authors was possibly due to previous systemic anti-mold therapy ^50^. In another study on 133 ICU patients, including 16 patients with proven or probable IA, who had a positive culture or GM test, the sensitivity and specificity of the LFD was 80% and 81% respectively ^51^. On the other hand, 18 patients without IA had a positive LFD result, although some of them grew mold in their respiratory specimens ^51^. There is no means to decipher between LFD and other microbiological criteria which results are false-positive or false-negative. This underlines the difficulties in assessing the performance of a new assay in the absence of contributive biopsies. To overcome some limitations of using LFD alone, a different study coupled qPCR and LFD and concluded to the high performance of this strategy, as in serum ^43^, even when the patients were given systemic anti-mold therapy ^52^. Therefore, as for GM or culture, an isolated positive LFD result in respiratory specimens could be limited to decipher between colonization and true infection, and the addition of several mycological tools could improve the reliability of IA diagnosis.

Exhaled volatile organic compounds—though not, strictly speaking, an antigen—have been investigated by electronic nose technology ^53^. *In vitro* investigations were conclusive to distinguish *A. fumigatus* from *Rhizopus oryzae*. The authors propose additional *in vivo* studies ^53^.

## Fungal identification in biopsies

Identification of species responsible for lesions in tissue is essential to adapt anti-fungal treatments. However, tissue is frequently processed for histopathology with formalin-fixed paraffin-embedded (FFPE) tissue, which hampers the molecular identification workflow because of technical issues ^54^. Fresh frozen tissue gives rise to a better yield than FFPE tissue in terms of qPCR detection as demonstrated in other molecular tests ^55– 57^. Once the DNA is extracted from biopsies, various strategies can be used, such as testing multiple species/genera by qPCR ^58^ or using sequencing of a pan-fungal barcode ^59^, microarrays ^60^, luminex-based methods ^61^, or PCR-electrospray ionization mass spectrometry ^62^.

However, pan-fungal primers can hybridize other eukaryotic DNAs. Therefore, the pan-fungal approach can be limited when tissue samples contain mainly human DNA, little fungal DNA, and potentially mixtures of fungi preventing sequencing of a single DNA barcode. The optimization of the pan-fungal primers is of utmost importance for a better representation of fungal species after PCR in complex media as in the current mycobiome studies ^63– 65^.

## New *Aspergillus* species and azole resistance

More and more, new mold species are reported as responsible for invasive infections. However, most of the reported cases are probable cases with isolation of these new species in respiratory specimens ^66^. Yet it is difficult to ascribe a new species to the pathology observed in the absence of biopsies confirming invasion. Indeed, a lot of different non- *Aspergillus* non-Mucorales species can be cultured from respiratory specimens in patients at risk of IA ^67, 68^. Identification still relies on microscopy and Sanger sequencing of some barcode genes, mainly Internal Transcribed Spacer (ITS) ^69, 70^, but it is expected that matrix-assisted laser desorption ionization-time of flight (MALDI-TOF) mass spectrometry can achieve a more rapid identification as the databases associated with the apparatus become more and more complete and reliable ^66, 71^. If these species emerge after or under azole therapy, a primary resistance is feared, such as for *Mucorales*, which is now more easily diagnosed using serum qPCR ^72, 73^. To know whether the new azole drug, isavuconazole, can have a specific role against these new species remains to be firmly established ^74^.

More challenging is the emergence of acquired azole resistance among *A. fumigatus* due to the wide use of azole drugs in agriculture and its therapeutic consequences ^75– 77^. The resistance mechanism is mainly mutation in the *cyp51A* gene associated with tandem repeats ^75^. It is not clear whether the resistant phenotype is associated with a higher mortality because of the resistant phenotype or other factors such as delayed diagnosis ^78^. Indeed, in a mouse model, the virulence of isolates with *cyp51A* mutations showed reduced virulence compared with azole-susceptible isolates ^79^, even if this lower virulence was not confirmed in *in vitro* models ^80^. However, when the resistance incidence in a particular setting is over 10%, one can challenge azole therapy as the first-line therapy ^81^. A consequence for the microbiological laboratory is the need to conduct surveillance of this resistance, at least the surveillance of the resistant phenotype ^18^. This surveillance can be completed by searching for the mutations responsible for azole resistance, either from colonies ^82^ or directly from the respiratory specimens ^83– 85^. Searching for other mechanisms of azole resistance is more difficult and these other mechanisms, in contrast to mutations in the *cyp51A* gene, could be associated with higher virulence ^80^. When PCR is used to directly detect mutations in respiration specimens ^83, 84, 86^, the difficulty is to know the different percentages of wild-type and resistant *Aspergillus* ^87^, given that both can be simultaneously present ^88^.

## Culture and next-generation sequencing

Given the uncertainties about biomarkers, culture remains an easy tool for the diagnosis of IA, as discussed earlier in ICU patients ^16^, and the detection of anti-fungal-resistant phenotypes. For the direct detection of resistance without culture, next-generation sequencing (NGS) will be more widely used to describe polymorphisms between isolates and mutations occurring during infection, in particular under anti-fungal treatments ^89^.

The main contribution of culture is not to focus on *A. fumigatus* but also to extend the spectrum to other mold infections ^67, 68^. NGS has been tested in the hope of dramatically extending this spectrum to every species present in the sample. A first strategy is to focus on ribosomal DNA (18S, 28S, or ITS) amplification using pan-fungal primers (metataxonomics) and to obtain operational taxonomic units to describe the diversity of the fungi in a clinical specimen. This strategy is dependent on the choice of the primers described above for the identification of mold in biopsies ^63– 65^. Indeed, with some primer sets, the results represent the more easily amplified species rather than the real content of the specimen.

Another strategy, called metagenomics, is to circumvent the amplification with predefined primers using whole genome sequencing. This needs extensive bioinformatic work to trim the many different sequences. This strategy is of interest if the goal is to correlate the proportion of bacterial and fungal organisms in samples ^90^ with a clear advantage on quantification compared with metataxonomics ^91^. This strategy is now compared in microbiology with culturomics ^92^. Culturomics consists of multiplying the culture conditions to detect low-growth microorganisms ^93^. Concordance between metagenomics and culturomics is not as good as expected ^93^, underlying the difficulties in detecting every microorganism using NGS. However, metagenomics seems more reliable than metataxonomics from the perspective of a diagnosis approach ^91^.

## Conclusions

The prognosis of IA is still dismal with an all-cause mortality around 40% at three months ^5, 32^, and emerging azole resistance puts even more pressure on favorable outcome ^78^. Stress is often put on the development of new diagnostic tools, but given the multiplicity of risk factors and the ubiquity of molds in the environment, there is little hope that these present microbiological difficulties will vanish in the near future. At the very least, a parallel stress should be put on the knowledge of the pathophysiology of the infection and the individual genetic susceptibility to infection. We found, for instance, evidence that the DNA detected using PCR is free circulating DNA ^94^. This has an immediate consequence for the pre-analytical step before nucleic acid analysis, although we cannot rule out that DNA also originates from other sources, such as fungal elements engulfed in circulating macrophages that require a different pre-analytical step to release fungal DNA from fungal elements.

For now, serum GM and DNA are the two most effective biomarkers for diagnosis, but their performance should be reappraised in new patient populations at risk of IA. Biomarkers in respiratory specimens also face difficulties in harmonizing clinical specimens. Biomarker evaluations will be hampered by the wide use of azole prophylaxis in hematology patients, which alters the kinetics of these biomarkers. This prophylaxis also exposes patients to IA due to resistant isolates and to other mold infections ^76^. Given the individual risk among a group of patients with similar treatments, the genetics underlying the susceptibility to IA should be further investigated to restrict the use of universal prophylaxis.
